# Retraction: FoxO3a Modulates Hypoxia Stress Induced Oxidative Stress and Apoptosis in Cardiac Microvascular Endothelial Cells

**DOI:** 10.1371/journal.pone.0344152

**Published:** 2026-03-04

**Authors:** The PLOS One Editors

After this article [1,2] was published, concerns were raised regarding results presented in Figs 2–3 and 5–7. Specifically,

- The following panels appear similar:
  - Fig 2A TUNEL 0h and Fig 2A TUNEL Sham in [3, retracted in 4], with cells removed.
  - Fig 2A TUNEL 2h and Fig 2A TUNEL 90 mg/kg in [3, retracted in 4], when flipped horizontally.
  - Fig 2A TUNEL 4h and Fig 2A TUNEL 60 mg/kg in [3, retracted in 4], when flipped horizontally.
  - Fig 2A TUNEL 6h and Fig 2A TUNEL 30 mg/kg in [3, retracted in 4], when flipped horizontally.
  - Fig 2A TUNEL 12h, Fig 3D SW480 10 µM in [5, retracted in 6], and Fig 2A TUNEL 10 mg/kg in [3, retracted in 4], when flipped horizontally.
  - Fig 2A TUNEL 24h and Fig 2A TUNEL Vehicle in [3, retracted in 4], when flipped horizontally.
  - Fig 2A DAPI 0h and Fig 2A DAPI Sham in [3, retracted in 4].
  - Fig 2A DAPI 2h and Fig 2A DAPI 90 mg/kg in [3, retracted in 4], when flipped horizontally.
  - Fig 2A DAPI 4h and Fig 2A DAPI 60 mg/kg in [3, retracted in 4], when flipped horizontally.
  - Fig 2A DAPI 6h, Fig 2A DAPI 12h, Fig 6A DAPI Control Crt siRNA in [7], Fig 2A DAPI 30 mg/kg in [3, retracted in 4], when flipped horizontally, and Fig 2A DAPI 10 mg/kg in [3, retracted in 4], when flipped horizontally.
  - Fig 2A ROS 24h, Fig 2A DAPI 24h, Fig 2A DAPI 24h in [7], Fig 2A Merge 24h in [7], and Fig 2A DAPI Vehicle in [3, retracted in 4], when flipped horizontally.
  - Fig 5A β-actin and Fig 5A β-actin in [8].
  - Fig 5A pFoxO3a and Fig 5A C-caspase-3 in [8].
  - Fig 6A Normoxia DAPI HIF siRNA, Fig 6A Hypoxia DAPI HIF siRNA, Fig 6B Hypoxia DAPI HIF siRNA, and Fig 6B Hypoxia Merge HIF siRNA.
  - Fig 8B TUNEL Hypoxia Crt siRNA and A2780 10 µM in part H in a figure in [9, retracted in 10].
- The following panels appear to partially overlap:
  - Fig 3A FoxO3a and Fig 5A β-actin.
  - Fig 3A Akt, GAPDH in a figure in [11] and Fig 5 BESA-2B NF-κB p50 (cytosolic) in [12, retracted in 13], and Fig 4 OE33 GAPDH in [14] when flipped vertically.
  - Fig 3A β-actin, Fig 5B β-actin in [8], and Fig 1 Calu-6 cells pentosidine Bcl-2 in [15, retracted in 16].
  - Fig 5A Akt and GAPDH in a figure in [11] when flipped horizontally.
  - Fig 7A FoxO3a and Fig 7A FoxO1.
  - Fig 7A BimEL and Fig 7A pFoxOa in [7].
- There appear to be vertical discontinuities in the following panels:
  - Fig 3A FoxO3a.
  - Fig 3A HIF-1α.
  - Fig 7A FoxO3a.
  - Fig 7A BimEL.

The authors did not respond to editorial request for comment on these concerns.

In light of the above concerns which call into question the integrity and reliability of [1,2], the *PLOS One* Editors retract this article.

All authors either did not respond directly or could not be reached.
